# The Potential Role of Kallistatin in the Development of Abdominal Aortic Aneurysm

**DOI:** 10.3390/ijms17081312

**Published:** 2016-08-11

**Authors:** Jiaze Li, Smriti Murali Krishna, Jonathan Golledge

**Affiliations:** 1 Queensland Research Centre for Peripheral Vascular Disease, College of Medicine and Dentistry, James Cook University, 4811 Townsville, Australia; jiaze.li@jcu.edu.au (J.L.); smriti.krishna@jcu.edu.au (S.M.K.); 2 Department of Vascular and Endovascular Surgery, The Townsville Hospital, 4811 Townsville, Australia

**Keywords:** kallistatin, serine proteinase inhibitors, abdominal aortic aneurysm, vascular remodelling, oxidative stress

## Abstract

Abdominal aortic aneurysm (AAA) is a vascular condition that causes permanent dilation of the abdominal aorta, which can lead to death due to aortic rupture. The only treatment for AAA is surgical repair, and there is no current drug treatment for AAA. Aortic inflammation, vascular smooth muscle cell apoptosis, angiogenesis, oxidative stress and vascular remodeling are implicated in AAA pathogenesis. Kallistatin is a serine proteinase inhibitor, which has been shown to have a variety of functions, potentially relevant in AAA pathogenesis. Kallistatin has been reported to have inhibitory effects on tumor necrosis factor alpha (TNF-α) signaling induced oxidative stress and apoptosis. Kallistatin also inhibits vascular endothelial growth factor (VEGF) and Wnt canonical signaling, which promote inflammation, angiogenesis, and vascular remodeling in various pre-clinical experimental models. This review explores the potential protective role of kallistatin in AAA pathogenesis.

## 1. Introduction

Abdominal aortic aneurysm (AAA) is usually defined as a permanent dilation of the abdominal aortic wall beyond a maximum diameter of ≥30 mm [1,2]. Progressive AAA dilatation can lead to rupture of the aorta, which causes bleeding and commonly death. AAAs most commonly affect men aged over 65 years [3], and clinical practice lacks effective treatment other than surgical approaches to repair AAAs [4]. Patients who have small AAAs (<55 mm), which are at low risk of rupture, are generally monitored through imaging surveillance. Patients with large (≥55 mm), rapidly growing (>10 mm/year) or symptomatic AAAs usually undergo repair by open surgical techniques or endovascular stents. However, postoperative morbidity and mortality are still common [2,5].

Studies of pre-clinical AAA animal models and biopsies of large human AAAs have implicated a range of mechanisms to be involved in the pathogenesis of AAA including degradation of the aortic extracellular matrix by a range of proteolytic enzymes, such as matrix metalloproteinases (MMPs); dysfunction of aortic vascular smooth muscle cells (VSMC) associated with their loss from the aortic media through apoptosis [6,7,8,9]; and inflammatory cells infiltration into the aortic wall which once activated produce pro-inflammatory cytokines, chemokines and proteolytic enzymes, which promote cell migration and vessel remodeling [2,10,11,12,13,14]. Other mechanisms implicated in AAA pathogenesis include angiogenesis [15] and oxidative stress [16]. AAA is often accompanied by atherosclerosis. This is in contrast to the aneurysms observed in genetic disorders, such as Marfan and Loeys-Dietz Syndromes.

Kallistatin is a member of the serine proteinase inhibitors (SERPIN) family. In human, it is encoded by the *SERPINA4* gene. It was first identified as a kallikrein binding protein that regulates the kinin-kallikrein pathway [17,18]. Kallikrein produces kinin from kininogens by proteolysis. Kallistatin binds to kallikrein to inhibit this process. Kallistatin has also been shown to have direct vascular effects, such as promoting vasodilation within rat models when human kallistatin is administered through gene overexpression [19]. Kallistatin is expressed in both endothelial cells (ECs) and VSMCs [20]. Kallistatin is also found in plasma, which is believed to reflect its production in the liver [17]. Decreased kallistatin levels have been previously associated with various disease conditions [21,22]. For example, Ma et al. reported decreased kallistatin level in the vitreous fluids in patients with diabetic retinopathy [21]. Zhu et al. reported decreased plasma kallistatin levels in apparently healthy African American adolescents with increased adiposity and cardio-metabolic risk [22].

Recent work has revealed potential protective functions of kallistatin in many pathophysiological processes implicated in AAA, such as inflammation [23,24,25,26], oxidative stress [25,27], angiogenesis [26,28,29], and hypertension [19,30,31]. The heparin binding domain of kallistatin is considered important for these functions [32,33,34]. Evidence from pre-clinical studies suggests that reducing inflammation [35], decreasing oxidative stress [36,37] and inhibiting angiogenesis [38] may limit AAA progression. Hence, in clinical management of AAAs, treatments targeting these mechanisms are considered to have potential benefits in managing AAAs [39]. In this review, we sought to highlight the potential regulatory roles of kallistatin in mechanisms relevant in AAA pathogenesis and also the downstream signaling pathways through which kallistatin exerts its actions.

## 2. Potential Roles of Kallistatin in AAA Pathogenesis

### 2.1. Kallistatin Attenuates Oxidative Stress

**Figure 1:**
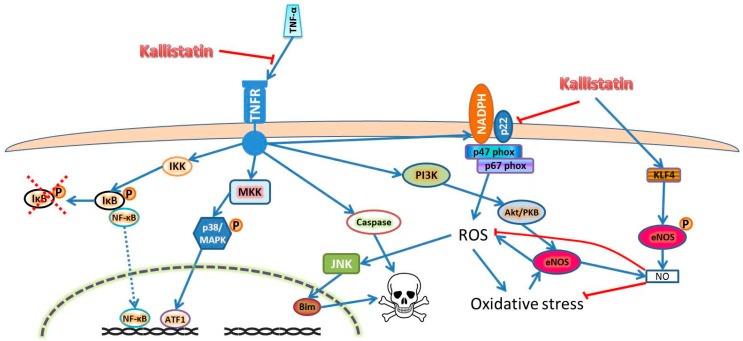
Kallistatin inhibits oxidative stress, inflammation and apoptosis through inhibiting TNF-α signaling and promotes NO production through eNOS stimulation. Kallistatin blocks TNF-α signaling through competitive binding to TNFR. This inhibits downstream signaling pathways that are activated by TNF-α, such as IκB/NF-κB and p38 MAPK pathway, which activate many pro-inflammatory and pro-angiogenic markers, such as TNF-α, VEGF, interleukins, MCP-1, MMPs and adhesion molecules. Kallistatin also inhibits TNF-α induces oxidative stress and the caspase cascade to induce apoptosis through TNFR-1. Alternatively, kallistatin is able to directly inhibit NADPH oxidase activity to attenuate ROS production, as well as activating eNOS through KLF4 to produce NO, which neutralizes ROS [43]. Abbreviations: endo-PC-endothelial progenitor cell, HUVEC-human umbilical vein endothelial cell, TNF-α-tumor necrosis factor alpha. Abbreviations: Akt/PKB—protein kinase B; ATF1—activating transcription factor 1; Bim—Bcl2 binding protein; eNOS—endothelial nitric oxide synthase; IκB—inhibitor of nuclear factor κ B; IKK—IκB kinase; JNK—c-Jun N-terminal kinase; KLF4—kruppel like factor 4; MAPK—mitogen activated protein kinase; MKK—MAPK kinase; NADPH—nicotinamide adenine dinucleotide phosphate; NF-κB—nuclear factor κB; NO—nitric oxide; P—phosphorylation; PI3K—phosphoinositide 3 kinase; ROS—reactive oxygen species; TNF-α—tumor necrosis factor alpha; TNFR—TNF-α receptor. The blue arrow lines indicate promotional activity; the red stop lines indicate inhibiting activity; the red dashed cross indicates degradation.

Tumor necrosis factor alpha (TNF-α) is a pro-inflammatory cytokine that has been consistently reported to be upregulated in AAAs [40]. TNF-α signaling initiates through binding of its membrane bound receptors TNFR-1 and 2. TNFR-2 is mainly expressed in immune cells and its functions remain unclear, while TNFR-1 initiates three major signaling pathways in cells, such as EC, as shown in Figure 1 [41,42]. Kallistatin has been shown to inhibit TNF-α induced oxidative stress and subsequent inflammation and apoptosis in experimental studies (Table 1) [25,27,43,44,45]. The inhibitory effects of kallistatin on TNF-α was discovered to be through competitive binding of TNF-α to the TNFRs through its heparin binding domain, thus inhibiting its signaling, which resulted in attenuated inflammation, oxidative stress and apoptosis of ECs [24,26,27].

Oxidative stress is caused by excessive production of reactive oxygen species (ROS). The ROS signaling pathway is also known as redox signaling [46]. High level of ROS have been shown to promote apoptosis of ECs, while continuous low level of ROS promote EC proliferation and migration that promote angiogenesis [47,48]. Nicotinamide adenine dinucleotide phosphate (NADPH) oxidase is the main source of ROS in ECs [46]. Interestingly, redox signaling and vascular endothelial growth factor (VEGF) signaling appear to be in feedback interaction in ECs [46,49]. Numerous stimuli are able to activate NADPH oxidase in ECs including VEGF, angiopoietin-1, angiotensin II, cytokines, shear stress and hypoxia [47,50,51].

There is a close relationship between oxidative stress and kallistatin activity. Oxidative stress has been shown to suppress circulating levels of kallistatin and EC specific expression of kallistatin [52,53], while kallistatin has been shown to suppress ROS production in cardiac and renal cells [45,54]. Many studies have suggested that kallistatin has anti-oxidative stress functions through inhibiting NADPH oxidase activities in various cell types, such as cardiac, epithelial progenitor cells (epi-PCs) and endothelial progenitor cells (endo-PCs), as well as in experimental models of myocardial infarction, hypertension and diabetes in rodents [44,54,55,56]. Furthermore, administration of anti-kallistatin antibody to rats has been reported to increase superoxide formation within the aorta and increased NADPH activity in the kidney and heart which eventually led to organ hypertrophy, inflammation and fibrosis. This was evidenced by a concomitant increased expression of pro-inflammatory genes such as *TNF-*α [25,54].

A study by Shen et al. reported that kallistatin attenuated aortic superoxide formation in salt induced hypertension in rats as well as inhibited TNF-α induced NADPH activity, oxidative stress and apoptosis through the PI3K-Akt-eNOS pathway in ECs [27]. ECs produce nitric oxide (NO) through endothelial nitric oxide synthase (eNOS), which neutralizes ROS. However, in oxidative stress conditions, the formation of peroxynitrite from superoxide and NO causes eNOS uncoupling and production of ROS [57,58]. ROS is known to induce apoptosis through inducing c-Jun NH_2_-terminal kinase (JNK) mediated Bim (a Bcl2 binding protein) nuclear translocation [53]. In an alternative pathway, kallistatin induces NO production through kruppel like factor–4 (KLF4) mediated eNOS activation and expression [43]. Thus, the switch of eNOS to produce NO by kallistatin stimulation inhibits ROS induced JNK-Bim mediated apoptosis [27]. Since ROS and cell apoptosis are implicated in AAA, stimulating kallistatin to upregulate NO production and limit cell apoptosis could be a potential target for therapy for AAA (Figure 1) [19,24,26,27,42,43,44,54,56,59,60,61,62,63,64].

### 2.2. Kallistatin Attenuates Angiogenesis and Inflammation

**Figure 2:**
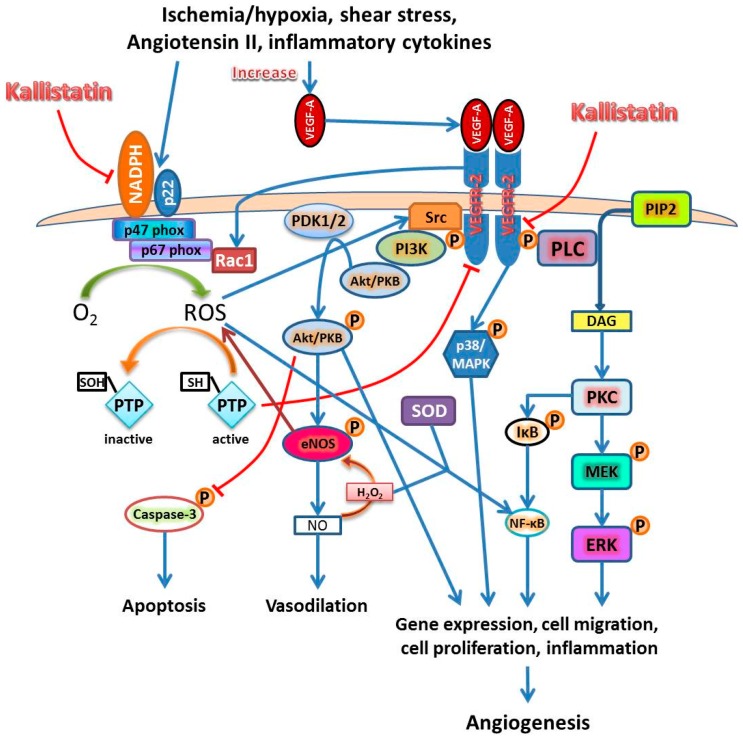
Kallistatin inhibits angiogenesis and inflammation through blocking VEGF signaling and NADPH oxidase activity. Kallistatin inhibits VEGF signaling through VEGFR-2 by its heparin-binding domain. VEGF-VEGFR signaling through PI3K-Akt pathway; the p38 MAPK pathway; and the PLC pathway leads to ROS/NO production, apoptosis, gene expression, cell migration, cell proliferation and inflammation. All of which are involved in angiogenesis. Kallistatin also directly inhibits NADPH oxidase activity and attenuates ROS production. NADPH oxidase is a complex consisting of several components. NADPH activity and VEGF-A/VEGFR-2 signaling have close interaction that is able to induce or activate many proangiogenic factors, such as MCP-1, VEGF, NF-κB, IL-8, VCAM-1, VE-cadherin and HIF1α in endothelial cells. Abbreviations: Akt—also known as protein kinase B—PKB; DAG—diacylglycerol; ERK—extracellular signal-regulated kinase; eNOS—endothelial nitric oxide synthase; HIF1α—hypoxia induced factor 1 alpha; IκB—inhibitor of nuclear factor κB; IL-8—interleukin-8; MAPK/MEK—mitogen activated protein kinase; MCP-1—monocyte chemoattractant protein-1; NADPH—nicotinamide adenine dinucleotide phosphate; NF-κB—nuclear factor κ B; NO—nitric oxide; PDK1/2—3-phosphoinositide dependent protein kinase 1 and 2; PI3K—phosphatidylinositol-3 kinase; PIP2—phosphatidylinositol 4,5-bisphosphate; PKC—protein kinase C; PLC—phospholipase C; PTP—protein tyrosine phosphatase; Rac1—small GTPase; ROS—reactive oxygen species; SOD—manganese superoxide dismutase; Src—non-receptor tyrosine kinase; VCAM-1—vascular cell adhesion molecule-1; VE—vascular endothelial; VEGF-A—vascular endothelial growth factor-A; VEGFR-2—VEGF receptor-2. The blue arrow lines indicate promotional activity; the red stop lines indicate inhibiting activity.

A previous study has shown that AAAs is associated with a marked angiogenic response directly related to the extent of inflammation within the aortic wall [65]. In this process, ECs proliferate and produce inflammatory cytokines, chemokines and MMPs. This initiates an influx of inflammatory cells which produce more cytokines, chemokines and MMPs that foster further endothelial activation, proliferation and inflammatory cell recruitment [66,67,68,69,70,71]. Upregulation of pro-angiogenic cytokines and medial neovascularization have been reported at the site of AAA rupture in human samples suggesting that angiogenesis plays an important role in AAA rupture [72]. VEGF is the most well-known and potent pro-angiogenic factor, especially the VEGF-A isoform [73,74,75,76,77]. There are three VEGF receptors—1, 2 and 3, identified so far. Among them, the type 2 receptor, VEGFR-2, which is also known as kinase insert domain receptor (KDR), a type III receptor tyrosine kinase, is the one that mediates downstream signaling of VEGF-A to induce EC activation and proliferation and promote angiogenesis (Figure 2) [29,32,44,46,48,54,56,77,78,79,80,81,82,83,84,85,86,87,88,89,90,91,92,93,94,95,96,97,98,99].

Kallistatin had been shown to inhibit VEGF signaling within in vitro studies. Huang and colleagues showed that recombinant human kallistatin inhibited VEGF165 mediated tyrosine phosphorylation of VEGFR-2 in human umbilical vein endothelial cells (HUVECs). Furthermore, it was also shown that the kallistatin mediated inhibition of VEGFR-2 was also accompanied by reduced downstream Akt and ERK phosphorylation [29]. The study reported by Miao and colleagues provided direct evidence of the ability of kallistatin to inhibit VEGF signaling by competitive binding to a VEGF receptor in human dermal microvascular endothelial cells (HDMECs). Using a site directed mutant of human kallistatin (K312A/K313A), they also confirmed that the heparin binding domain of kallistatin is important to this function [34].

**Figure 3:**
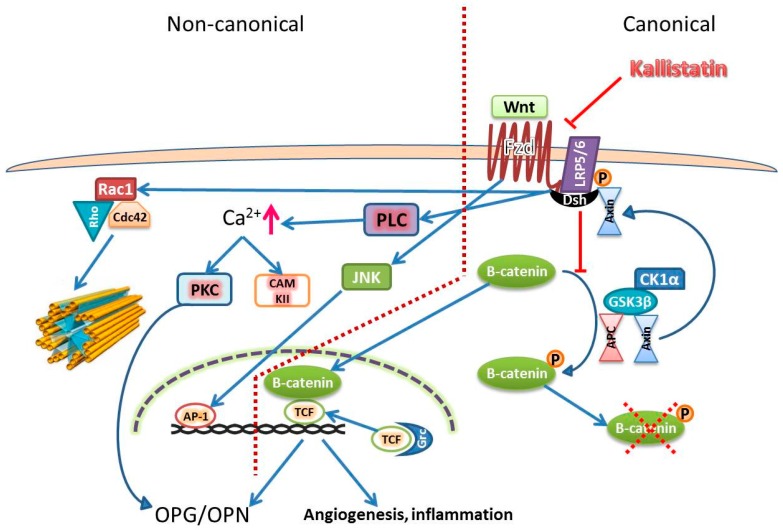
Kallistatin inhibits Wnt canonical pathway induced angiogenesis and inflammation. In the Wnt canonical pathways, mostly mediated by Wnt1, 3, 3a, 7a and 7b, Wnt/Fzd binding phosphorylates the associated co-receptor LRP5/6. This recruits Dsh which leads to binding of Axin at the membrane. Axin forms a degradation complex with APC, CK1α and GSK3β for β-catenin degradation. The recruitment binding of Axin to the membrane caused by Wnt/Fzd leads to an inactive degradation complex and the accumulation of β-catenin. The accumulated β-catenin mediates Wnt signaling by activating transcription factors, such as TCF, which induces transcription of genes, such as VEGF, ICAM-1 and TNF-α. Kallistatin binds to LRP6 and prevents LRP6 from phosphorylation which results in β-catenin degradation. Without β-catenin, Wnt canonical signaling is blocked. Abbreviation: AP-1—activator protein-1; APC—adenomatous polyposis coli; CAMKII—calmodulin dependent protein kinase; CK1α—casein kinase 1α; Dsh—the protein disheveled; GSK3β—glycogen synthase kinase-3β; ICAM-1—intracellulcar adhesion molecule-1; LRP5/6—low density lipoprotein receptor-related protein 5 or 6; PCP—planar cell polarity; OPG—osteoprotegerin; OPN—osteopontin; TCF—T-cell factor; TNF-α—tumor necrosis factor alpha; VEGF—vascular endothelial growth factor; the red arrow indicates increase in level; the tubular structure on the left represents cytoskeleton. The blue arrow lines indicate promotional activity; the red stop lines indicate inhibiting activity; the red dashed cross indicates degradation.

The Wingless (Wnt) signaling pathway is a tightly regulated, highly complex system which mediates a diverse range of cellular activities including proliferation, apoptosis, migration and differentiation, all of which are relevant in AAA pathogenesis. There are 19 potential Wnt ligands that are able to bind to 10 transmembrane G-protein-coupled receptors of the Frizzled (Fzd) family [100]. The signaling pathways activated as a consequence of Wnt/Fzd binding are categorized into canonical and non-canonical pathways (Figure 3) [101,102,103,104,105,106,107,108]. In vitro experiments suggest that kallistatin inhibits the Wnt pathway at the extracellular or the receptor level. Kallistatin binds to the extracellular domain of low density lipoprotein receptor-related protein 6 (LRP6) which blocks Wnt canonical pathway signaling through β-catenin [105,109,110]. This has potential anti-angiogenic and anti-inflammatory effects as shown by Liu et al. in a diabetic mouse model of retinopathy [105]. It was shown that overexpression of human kallistatin in the retina of Akita mice, significantly decreased the expression of pro-angiogenic factors such as VEGF, intercellular adhesion molecule (ICAM)-1 as well as the number of CD11^+^ b leukocytes suggesting that overexpression of kallistatin suppressed Wnt signaling induced by ischemia or diabetes [105]. They also demonstrated that human kallistatin reduced VEGF and TNF-α levels which were increased in retinal cells treated with high glucose in culture [105]. Similar phenomenon of attenuated VEGF and TNF-α production by kallistatin were also observed in breast cancer and wound healing models [110,111].

The Wnt non-canonical pathway is mostly mediated by Wnt4, 5a and 11 resulting in increased Ca^2+^ which activates PKC and CAMKII which often activate nuclear factor of activated T-cells that promotes VEGF induced angiogenesis. Another signaling pathway activated by the Wnt non-canonical pathway is JNK which leads to gene transcription by activating AP-1. The Wnt non-canonical pathway is also able to activate the PCP pathway which leads to cell polarization and cytoskeletal rearrangement in ECs.

A number of preclinical studies have suggested that kallistatin had anti-angiogenic functions (Table 2) [26,28,105,112,113]. In animal models of diabetes or oxygen induced retinopathy and neovascularization, administering human kallistatin to retinal cells or overexpressing human kallistatin in transgenic mice ameliorated neovascularization through inhibiting VEGF activity, endo-PC release from bone marrow and reducing activation of the Wnt canonical pathway [105,112,113]. The Wnt canonical pathway has been shown to stimulate EC proliferation and survival through VEGF-A upregulation [104,114]. In addition, Wang and colleagues reported that kallistatin inhibited proliferation of HDMECs and reduced vessel density in the ankles of arthritic rats through reducing TNF-α [26]. TNF-α was previously shown to induce the gene expressions of VEGF-A, VEGFR-2 and its co-receptor neuropilin-1 [60]. Further to this, kallistatin was shown to inhibit the Wnt canonical pathway through binding to LRP6 and inhibiting TNF-α in cancer cells, both of which also resulted in reduced VEGF expression [28,110]. Thus, it is evident that kallistatin has anti-angiogenic effects.

### 2.3. Kallistatin Attenuates Defective Vascular Remodeling

Vascular remodeling is a dynamic process that changes the structure of blood vessels to maintain a healthy state however in excess it contributes to AAA formation. Over activation of several cellular activities including apoptosis, proliferation, migration and degradation of the extracellular matrix contribute to excess vascular remodeling [115]. TNF-α is involved in all these four processes through inducing production of VEGF, interleukins, cellular adhesion molecules and MMPs. The ability of kallistatin to inhibit TNF-α and thereby limit angiogenesis, apoptosis, oxidative stress, inflammation, and cell proliferation and migration may ameliorate defective vascular remodeling and may play a protective role in vascular disorders, such as AAA. Kallistatin was originally shown to inhibit kallikrein and thereby limit kinin formation [17,18]. Kinins have been implicated in AAA formation within rodent models. Kinin B2 receptor blockade has been reported to protect against AAA development, growth and rupture in a mouse model, as well as reducing MMP secretion from human AAA explant in vitro [116]. Kinin B2 receptor blockade also limited neutrophil activation and development of an inflammatory phenotype in VSMCs in vitro. The inhibitory effect of kallistatin on tissue kallikrein would be expected to limit kinin generation and thereby antagonize the pro-aneurysmal effects of the kallikrein-kinin pathway. However, there is evidence suggesting that kallistatin also increases MMP-2 activity in human endo-PCs through enhancing NO and VEGF levels by activating PI3K-Akt signaling [44]. Although this may facilitate vascular repair and regeneration through promoting migration of endo-PCs. MMP-2 activity also contributes to medial matrix degradation in AAAs and possibly even rupture [9]. Thus, the protective role of kallistatin in maintaining positive vascular remodeling remains to be investigated further in pre-clinical AAA models.

## 3. Conclusions

**Figure 4:**
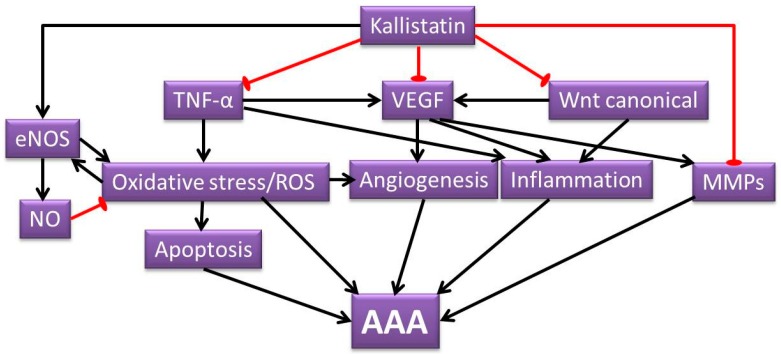
Illustration of postulated mechanisms of kallistatin attenuating AAA. Kallistatin has the potential of inhibiting various mechanisms that contribute to AAA formation. The pathological processes that are attenuated by kallistatin include oxidative stress, ROS signaling, apoptosis, angiogenesis, inflammation and MMP activity. The proposed AAA protective role of kallistatin are through its ability to inhibit various pathways, such as TNF-α, VEGF and Wnt canonical signaling pathways, as well as kallistatin’s ability to increase NO production through eNOS. The black arrow lines indicate promotional activity; the red stop lines indicate inhibiting activity.

In summary, AAA is a vascular disorder that is characterized by inflammation, apoptosis and extracellular matrix degradation. This review illustrates the potential of kallistatin in suppressing AAA development through attenuating a wide range of pathological mechanisms (Figure 4) including VEGF induced angiogenesis and inflammation, oxidative stress induced angiogenesis and apoptosis, TNF-α induced inflammation, apoptosis and MMPs production, as well as Wnt canonical signaling induced angiogenesis and inflammation. Direct studies examining the role of kallistatin in AAA are warranted and also should assess the potential beneficial effect of kallistatin. Since kallistatin has a range of actions, some of which may be detrimental, as well as beneficial, future studies should consider both systemic and local upregulation of kallistatin. Achieving elevated kallistatin levels through recombinant protein delivery and transgenic overexpressing methods has been reported to reduce blood pressure in animal models [19,31,117], which could be beneficial in treating AAA patients. However, this warrants further preclinical studies using established AAA models.

## Figures and Tables

**Table 1:** Studies showing the inhibitory effects of kallistatin mediated through blocking TNF-α signaling on pathologies relevant to abdominal aortic aneurysm such as oxidative stress, inflammation and apoptosis.

Inhibited Pathology	In Vitro Model	In Vivo Model	References
Oxidative stress/inflammation	Proximal tubular cells, mesangial cells	Dahl-salt sensitive rats	[45]
	HUVEC	–	[43]
	–	Hypertensive rats	[25]
Oxidative stress/apoptosis	Rat and human endo-PC	Deoxycorticosterone acetate salt-hypertensive rats	[44]
	HUVEC	Rats	[27]

Abbreviations: endo-PC—endothelial progenitor cells; HUVEC—human umbilical vein endothelial cells; TNF-α—tumor necrosis factor alpha.

**Table 2:** Studies assessing the inhibitory effects of Kallistatin mediated through blocking VEGF, TNF-α and Wnt canonical signaling pathways on pathologies relevant to abdominal aortic aneurysm such as angiogenesis and inflammation.

Inhibited Pathology	Pathways	In Vitro Model	In Vivo Model	References
Retinal neovascularisation/angiogenesis	VEGF	Retinal capillary ECs	Brown Norway rats	[112]
Angiogenesis in cancer	TNF-α/VEGF	MCF-7 cells, HUVEC	Fertilized chicken egg	[28]
Angiogenesis/inflammation arthritis	TNF-α	HDMEC	Rats	[26]
Angiogenesis/Inflammation Diabetic or OIR	Wnt canonical pathway	Retinal cells	Kallistatin transgenic mice with OIR or type I diabetes	[105]
Oxygen induced retinopathy/angiogenesis	Wnt canonical pathway	–	Kallistatin transgenic mice, bet-gal mice	[113]

Abbreviations: EC—endothelial cells; HDMEC—human dermal microvascular endothelial cells; HUVEC—human umbilical vein endothelial cells; MCF—Michigan Cancer Foundation (MCF-7 is a breast cancer cell line); OIR—oxygen induced retinopathy; TNF-α—tumor necrosis factor alpha; VEGF—vascular endothelial growth factor.

## Abbreviation

AAA	abdominal aortic aneurysm
eNOS	endothelial nitric oxide synthase
MMPs	matrix metalloproteinases
NO	nitric oxide
ROS	reactive oxygen species
TNF-α	tumor necrosis factor alpha
VEGF	vascular endothelial growth factor
